# The US Virtual Herbarium: working with individual herbaria to build a national resource

**DOI:** 10.3897/zookeys.209.3205

**Published:** 2012-07-20

**Authors:** Mary E. Barkworth, Zack E. Murrell

**Affiliations:** 1 Intermountain Herbarium, Department of Biology, Utah State University, 5305 Old Main Hill, Logan, Utah, USA 84322-5305; 2 Biology Department, Appalachian State University ASU Box 32027, Rankin Science Building, 527 Rivers Street, Boone, North Carolina, USA 28608

**Keywords:** Herbaria, networks, plants, fungi, algae, digitization, online databases

## Abstract

The goal of the US Virtual Herbarium (USVH) project is to digitize (database, image, georeference) *all* specimens in all US herbaria, enabling them to be made available through a single portal. Herbaria house specimens of plants, fungi, and algae, so USVH will offer a rich portrait of biodiversity in the US and in the other countries represented in US herbaria. Equally importantly, working towards this goal will engage people with herbaria and the organisms they house, expanding their appreciation of both the power of biodiversity informatics and the demands that it places on data providers while developing improved communication among those working in and with herbaria. The project is not funded but has strong support among those working in herbaria. It works through regional herbarium networks, some of which existed prior to the USVH project, while others are still in gestation. It differs from most digitization projects in its emphasis on helping those involved with herbaria become part of a national enterprise, an aspect that is seen as critical to creating the resources needed to develop and sustain the project. In this paper, we present some of the lessons we have learned and the difficulties we have encountered during the first few years of the project.

## Origin of the US Virtual Herbarium project

The US Virtual Herbarium project was started in 2008 at a meeting held in conjunction with the annual meeting of the Botanical Society of America. Those present were asked whether they were in favor of attempting to develop integrated access to specimen information residing in all US herbaria, creating in essence, a US Virtual Herbarium (USVH). The meeting followed 20+ years of digitizing efforts (primarily databasing) within US herbaria. It had been called because, despite these efforts, there was no evidence of a program to build a national resource that would include all herbaria. Some of those voting had been involved in digitization efforts. Others came looking for help, both financial and technical, in starting the process. At the end of the meeting, all those present endorsed the concept. Thus the project started, not in direct response to a national initiative or program but as a statement of interest by those directly involved with herbaria.

The meeting was held under the auspices of the Western Association of Agricultural Experiment Station Directors (WAAESD). Each state has an Agricultural Experiment Station (AES) and their directors work together, regionally and nationally, in areas of joint interest. Although it was AES directors in the western states who sponsored the meeting, the USVH project has always been national in scope. Formally speaking, the purpose of the meeting was to determine whether there was sufficient support to justify WAAESD sponsorship of a 5-year committee to coordinate work towards a single access point to information from all US herbaria. Given the support expressed, formation of the committee was approved.

WAAESD sponsorship provides a formal but flexible structure within which to operate. It does not provide funding; it does provide freedom in determining how best to pursue a group’s objectives. It also provides a mechanism for disseminating information through the National Information Management and Support System (NIMSS). Reports and announcements posted to NIMSS are sent to AES directors in each state as well as to registered participants. Because most herbaria are not connected with AES, the sponsorship by WAAESD immediately increased awareness of herbaria.

The executive committee’s first task was to develop explicit goals for the project. After considerable debate, it agreed that the overall goal of the US Virtual Herbarium project should be digitizing all specimens in all US herbaria. The result will be a major new scientific resource but the greatest benefits will result from working towards this overall goal, a process that will require helping collectors and curators record information in a manner that maximizes the value of a specimen, use the tools being developed for capturing and sharing collection information, and make use of the resulting information in their research, education, and outreach activities. It will also require increasing interaction among those who work in herbaria and educating users in diverse disciplines about the value and use of collection data. Much of the value of the project lies in ensuring that these benefits are experienced by all those involved with herbaria and in teaching students about algal, fungal, and plant diversity.

Herbarium specimens provide a particularly rich information layer to the world’s biodiversity resources because they represent sessile organisms. They show the ability of a taxon to complete its life cycle at a particular location and time and, in some instances, provide information about the prevailing growing condition (see, e.g., Woodward and Bazzaz 1988; Kouwenberg et al. 2003; Zangeri and Berenbaum 2005; Johnson 2011). Thus the value of the digital herbarium layer is clear. The optimal path (or paths) to providing it is less clear. The task of the US Virtual Herbarium project is to accelerate the process and ensure that all herbaria become involved because in that way more individuals will learn about the organisms present in herbaria, what digitization involves, and the power of biodiversity informatics. It will also result in a more dense information layer. The project does not focus on developing better ways to digitize herbaria; that is the focus of specific programs within the National Science Foundation and Institute for Museum and Library Services. Instead, the project aims to foster the collaborations needed to establish networks and enable rapid dissemination of better procedures as they become available. In this paper, we share some of the lessons we have learned in reaching the current level of digitization in the US.

## Herbaria in the US

**Figure 1.**
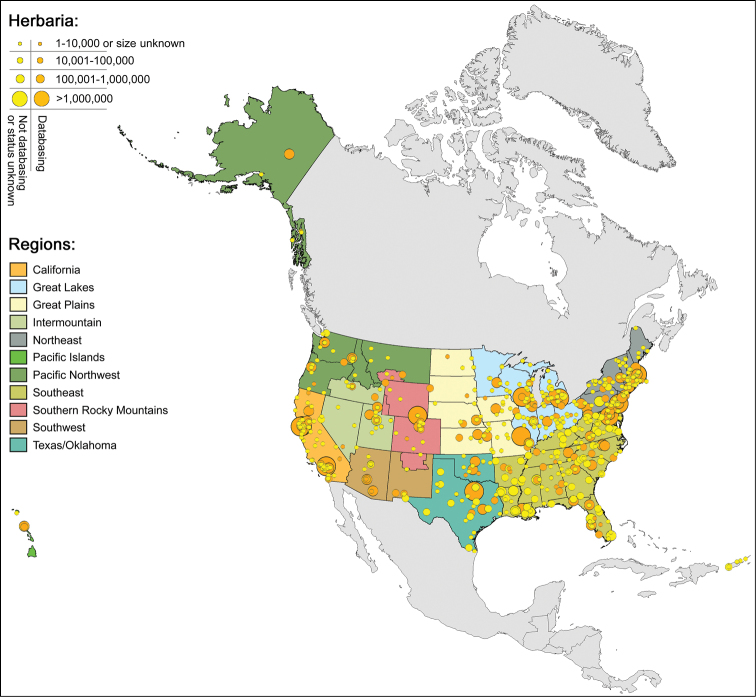
Regional networks and herbaria in the U.S.A. Network boundaries are guides; herbaria are free to join the network of their choice. Some herbaria contribute records to more than one network. No network has been established as yet for the Great Plains, Great Lakes, and Southern Rocky Mountain Regions. Data obtained June, 2011.

There are 729 registered herbaria in the US (Thiers et al. 2012+). They are scattered throughout the country but are more abundant in densely populated states (Fig. 1). Seventeen herbaria have a million or more specimens each; about 300 have fewer than 17,000 specimens. About 150 of the US herbaria listed in Thiers et al. (2012+) have been transferred or closed; there are also many herbaria not listed by Thiers et al. (2012+), most of which have fewer than 10,000 specimens. Our current estimate is that there are about 800 active herbaria and over 90 million herbarium specimens in the US.

About 78% of US herbaria are owned by an academic institution. Academic herbaria, particularly those in smaller institutions, offer excellent opportunities for involving students. Countering this potential is the fact that small herbaria often receive little or no formal support from their institution and may not be actively curated. Of the remaining herbaria, about 13% are owned by a government entity, usually federal but in some cases state, county, or municipal. About 9% are associated with botanical gardens or independent museums; among these are eight of the herbaria with a million or more specimens.

In 2009, Thiers provided Barkworth with a list of US herbaria registered with *Index herbariorum* at that time. Of these, 601 appeared to be active. “Appeared to be” because there is no guarantee that Thiers is notified when a herbarium is closed or transferred. In 2010 a survey (via paper questionnaire, with reminders by email or telephone call to some non-respondents; see Appendix 1) of all 601 herbaria resulted in 287 responses (Barkworth 2011, unpubl. data). The data revealed that many of the smaller, non-responding herbaria had been transferred or closed. Of the responding herbaria, 154 (54%) had a herbarium database and 70 (24%) were imaging their specimens. Collectively, the 287 herbaria held 50,583,000 specimens, of which 16,880,000 (33%) had been databased and 1,510,000 (3%) imaged. Most of the databasing herbaria (150/154) made specimen information available on the web through their own web site; 39 did so through a regional website; 38 made their records available to the Global Biodiversity Information Facility. These data indicate strong commitment to digitization and data sharing among US herbaria.

In addition to there being many herbaria in the US, there are many different taxonomic opinions, particularly with respect to vascular plants. These are reflected in state and regional floras. There are resources to help interpret the resulting complexity, e.g., *Flora of North America* (FNA; Flora of North America Editorial Committee 1993-present), which is developing a single taxonomic treatment for all bryophytes and vascular plants in North America north of Mexico. These are not always accepted but Tropicos (http://www.tropicos.org/, see the list of relevant websites in Appendix 2) shows how different floristic treatments have treated a particular name. *Index fungorum* (http://www.indexfungorum.org/names/names.asp), and Algaebase (http://www.algaebase.org/) are internationally respected indices to fungal and algal names, respectively. The US Virtual Herbarium project accepts that records in different herbaria may reflect multiple taxonomic concepts, a reality that can only partially be accommodated by alternative tables of synonyms. There are undoubtedly instances where this creates problems, for instance, when interpreting the distribution of a taxon that is sometimes interpreted narrowly, sometimes broadly, but such situations are probably less common than problems caused by misidentifications.

Table 1 shows the current status of herbarium digitization in the US from a network perspective. The six existing regional networks involve about 200 herbaria, ranging from small, unlisted herbaria to the largest herbaria in the country. Some herbaria contribute to multiple portals. The number of records available is over 7,665,000. This count does not differentiate between those that are fully databased, imaged, and georeferenced and those that have minimal information, possibly only the image of a label. Progress in the different aspects is hard to assess. Only the Pacific Northwest Herbaria (PNW) portal shows the number of specimen images available and only Symbiota portals show how many records have georeference data. Many georeferenced records do not include uncertainty estimates. The California, Pacific, and Pacific Northwest networks use software developed within each region; the portal for the southeastern US uses a mixture of software; the others use Symbiota (http://symbiota.org).

**Table 1.** Overview of US regional and taxonomic herbarium networks. The Southwest and Intermountain Regions share a database but have different portals. “Herbaria” indicates the number of herbaria currently providing information to the network; numbers in parentheses are for extra-regional herbaria. Records are text-based records. Geo: percentage of georeferenced records. Most data obtained from web sites or node managers, March 31, 2012

Network	URL	Taxonomic scope;Location of source herbaria	Herbaria	Records
Existing networks				
California herbaria (CA)	http://ucjeps.berkeley.edu/consortium	Vascular plants;California	20 (1)	1,454,000
Pacific Northwest Herbaria (PNW)	http://www.pnwherbaria.org	US: Alaska to Oregon + Idaho and Montana. CANADA: British Columbia, Yukon.	57	1,763,040 (174,160 images)
Southwest (SEINet) and Intermountain (IRHN)	http://swbiodiversity.org/seinet/index.php(Shared database; different portals)	US: Southern California east to New Mexico, north to Nevada, Idaho, and ColoradoMEXICO: Baja California, Sonora;Vascular plants.	32 (2)	2,069,025(67% Geo)
Pacific Islands (CPH)	http://www.herbarium.hawaii.edu/cph/index.html	Hawai’i and the Pacific basin [Currently 3 of 15 herbaria connected]Vascular plants.	15	60,000
Northeast (CNH)	http://neherbaria.org/CNH	US: north and east from Pennsylvania CANADA: Ontario eastward;All taxa.	58	409,883
Southeast(SERNEC)	http://www.herbarium.unc.edu/seflora/firstviewer.htm	From Eastern Texas to Virginia to the Atlantic and Gulf Coasts;All taxa.	14	140,000
Wisconsin Flora	http://www.botany.wisc.edu/wisflora/	Wisconsin;Vascular plants, lichens	8	370,000
Alabama Plant Atlas	http://www.floraofalabama.org	Alabama;Vascular plants	9	78,000
Bryophytes	http://symbiota.org/bryophytes/index.php	North America;Bryophytes.	10	922,047(38% Geo)
Lichens	http://symbiota.org/nalichens/index.php	North America; Lichens.	16(1)	627,756(55% Geo)
Macrofungi	http://mycoportal.org/portal/index.php	North America; Macrofungi	5	154,526(13% Geo)
American Myrtaceae	http://cotram.org	Myrtaceae in the Americas	4	64158 (63%)

## Lessons learned

• Commitment, energy, time, resources, and funding are the most critical needs of the USVH project. Of these, time is usually the most scarce resource, particularly in smaller herbaria in which a single individual has to fulfill many different functions. It can, of course, be alleviated to some extent by funding but digitization will require a time commitment on the part of the person or persons responsible for a herbarium. Funding for other resources is also needed but much can be done with minimal financial support now that effective software and work flows have been developed, particularly if hardware is shared.

• The range in size of US herbaria (from less than 1000 to over 8 million) and their diverse roles is matched by the diversity of their resources and goals. Many have little or no IT support and little or no budget; others, even some smaller herbaria, have strong IT support, significant endowments, and substantial volunteer support. Goals range from research on a global level to being a reference collection for training of seasonal employees.

• Curators have diverse backgrounds. Most, particularly in mid-sized to large herbaria, are professionally trained taxonomists with memberships in professional societies such as the Botanical Society of America and the American Association of Plant taxonomists. Others have backgrounds that range from ecology to paleobotany, with their professional associations being equally diverse. This presents a challenge to developing an effective information flow among all herbaria. Regional collaborations on multiple scales are effective in addressing this challenge but require a leader with time to commit to the task.

• There is no best approach for digitizing herbaria; there are multiple effective approaches. The needs and resources of large research herbaria with multiple type specimens and collections from many countries and multiple centuries differ from those of small herbaria serving a forest district or a teaching institution. In working with those in charge of herbaria, one must recognize and respect their differing priorities and resources. Adopting theoretically suboptimal procedures for digitization may be the best procedure if the resources needed for adopting a better procedure are not available.

• Broadening participation requires minimizing barriers while maximizing benefits. Symbiota (http://symbiota.org/tiki/tiki-index.php), open source software available through SourceForge (http://sourceforge.net), accomplishes this by enabling direct data entry into the central database, providing tools for preparing labels, and integrating images of living organisms into checklists, species pages, and flash card quizzes. In August 2011, Barkworth switched the Intermountain Herbarium (258,000 specimens) to databasing directly into the regional database (SEINet/IRHN) which uses Symbiota. It was so easy to use that she persuaded two colleagues, Gordillo and Anderson, each of whom is responsible for a small herbarium (6000 and 4000 specimens, respectively), to employ it to bring their herbaria into the network. The financial cost for the two was less than $400 each, the cost of preprinted barcode labels and a barcode scanner. Data entry is being done by volunteers. Of equal importance, students introduced to the program and associated portal immediately see value in the resources provided. Once imaging equipment is available, the two herbaria will adopt procedures that exploit the advantages images offer but, in the meantime, their students are learning to record better information and their institutions can boast about contributing to a major resource.

• It does not matter whether a herbarium starts with imaging or databasing. The important thing is to start. Specimen records that consist only of text-based information can be used for generating checklists, georeferencing, and searching. Specimen records that consist only of an image are of little value until the label information is databased but imaging can accelerate databasing and enable offsite-databasing. Establishing both of these, however, requires infrastructure development, both technical and human.

• Remote data entry and incorporation of optical character technology into the data entry process can speed up data entry but it requires access to images which, in turn, requires access to appropriate equipment. Legler (2011) has designed equipment that has been widely adopted because it is effective, easily transported, and does not take much space. The problem is that the initial cost (about $6000) is large compared to the budgets of most herbaria. Once purchased, it can be shared among neighboring herbaria, a process that also fosters the kind of social network needed to disseminate information.

• Integrating optical character recognition (OCR) technology into data entry tools will accelerate data entry for the very large number of specimens with clean, typewritten or computer generated labels but entries need to be reviewed before being accepted. Major obstacles to widespread adoption of OCR-assisted data capture are a) lack of imaging equipment and b) the need to incorporate OCR-assistance into the data entry module of the various database systems used in herbaria, a process that is underway. For interpreting hand-written or unclear labels, OCR is less effective than humans.

• Automated georeferencing tools, such as Geolocate (http://www.museum.tulane.edu/geolocate/) greatly accelerate georeferencing and can provide an estimate of uncertainty but, as with OCR data entry, the results, both for the locality and the uncertainty, need to be reviewed. At present, most programs for sharing information can only store point-radius uncertainties, not a polygon. This limits their value because plant collectors often collect along a trail. Another potential problem is that all values are calculated based on current geographic information. Even with such limitations, georeferencing is valuable. Applied to the thousands of specimens in herbaria, it enables patterns to be detected even if some of the individual locations are fuzzy. Those using the data should be aware of the inherent problems, grateful for the amount of data being provided, and willing to assist in improving its quality.

• Batch georeferencing, in which multiple specimens with the same locality information are georeferenced simultaneously, greatly accelerates georeferencing. The acceleration is greatest if records from multiple herbaria can be georeferenced simultaneously. Technological impediments to effective batch georeferencing include the absence of a mechanism for sharing specimen records among networks and the need for tools that “repatriate” the georeferencing information back to the specimen records. The human impediments include lack of knowledge as to how to georeference specimens and/or use the tools available for assisting in the task, impediments that can be overcome by workshops and online tutorials. Another impediment is the need for effective management of such collaborations.

• Enabling collectors to enter their collection information directly into a database that can both generate labels and provide data to the databases of recipient herbaria should be given high priority. Ideally, such programs should make it possible to enter information whether offline or online and for multiple taxonomic groups because individuals frequently collect more than one kind of organism. If data are entered offline, it should be possible to clean them when the connection is restored. (see, e.g., Atrium http://www.atrium-biodiversity.org/about.html). Label-making modules should also enable students to use the module while taking a class without the data being displayed so that they learn to record and store data in a manner that maximizes its utility.

Label generating tools will not help digitize the specimens currently in herbaria but early adoption of database-driven label production combined with aggressive pursuit of funding opportunities enabled the herbaria of the University of Wyoming and the Missouri Botanical Garden (1.4 and 6.3 million specimens, respectively) to have over 50% of their collections databased by the time of the survey. The only other large US herbarium to have more than 50% of its 950,000 specimens databased is the National Fungus Collection which has 89% of its collections databased, a noteworthy accomplishment.

• Regional collaborations are the most effective method of spreading digitization. They make it easier to share imaging equipment and develop the localized resources (e.g., checklists, identification tools) that give immediate, easily recognized value to regional portals. They also make establishing personal relationships among data providers easier, relationships that subsequently become effective social networks for sharing ideas and information. Development of regional networks is also critical to building the long term, broadly based support required to create and sustain a truly national herbarium network, one that involves all herbaria.

• The map (Fig. 1) shows the major regional networks but there are many smaller digitization networks in existence, some of which were initiated with federal funding, others with state or private funding. They have been critical to bringing the digitization of US herbaria to its present status. These smaller networks generally make their records available through their own web site. One of the challenges facing the US Virtual Herbarium project is to enable such networks to share their specimen information more widely. Other challenges include establishing networks for all parts of the country and persuading herbaria with their own web site to share their records on a regular basis with a regional network.

• There is often a lag time between agreeing to establish a network and actually having a network that people can use. Herbaria with their own specimen databases need to develop scripts for exporting their data to the network database and ensuring that new and modified records are exported at regular intervals. Constructing and testing these scripts takes time. It may also be found that the existing data has to be cleaned up before being exported. Another source of delays can come from establishing formal memoranda of understanding. Delays are greatest if the herbaria are located in different countries or belong to a private institution. Some networks operate without formal memoranda.

• There is a need for the single, all-embracing network that is being established by iDigBio (see below). At present, herbaria with specimens from different taxonomic groups need to send their data to multiple networks (there are separate networks for bryophytes, lichens, and macrofungi). Moreover, at present regional nodes only provide access to specimens from herbaria within their region, e.g., data for specimens from the northeastern US residing in herbaria of the intermountain region are not currently made available to the northeastern network. It also means that users wishing to examine all biodiversity within a region have to go to multiple networks to obtain the information they seek and each network. To maximize the value of a truly integrated network, however, its data must be readily accessible and easily queried not just by biodiversity informatics specialists but also by the general public and educators at all levels and in many different disciplines because it is, ultimately, these people whose support will be required to sustain the network’s maintenance and development.

## Interaction with iDigBio and BISON

In February 2010, an NSF-funded workshop brought together individuals with knowledge in different aspects of digitization to discuss how best to develop a national herbarium network. Several useful discussions and contacts resulted from the workshop but that fall the NSF announced its Advancing Digitization of Biological Collections (ADBC) Program. ADBC projects fall into two categories, creation of “a permanent database of digitized information from all biological collections in the U.S. (https://www.iDigBio.org/content/about-iDigBio)”, the iDigBio project, and Thematic Collection Networks (TCNs) that focus on “major scientific questions” (http://www.nsf.gov/news/news_summ.jsp?cntn_id=121015). At about the same time it was announced that what is now the Biological Informatics Program of the US Geological Service had begun development of an integrated and permanent resource for biological occurrence data from the United States, Biodiversity Information Serving Our Nation (BISON). This will integrate records for the US from the Global Biodiversity Information Facility and those made available via iDigBio with multiple geographic environmental layers, thereby enabling sophisticated and complex analyses.

These two developments forced us to rethink how the US Virtual Herbarium project could best achieve its objectives, assuming they were still valuable, while complementing the work of ADBC-funded projects. The goals of the US Virtual Herbarium project are similar to those of iDigBio apart from its sole focus on herbaria, but it has a somewhat different emphasis. For iDigBio, extending participation to all collections in the US, both large and small, is a third phase, while for USVH, it is the priority. A recent analysis of the botanical capacity of the US (Kramer et al. 2010), demonstrated that the country has far fewer students entering the botanical sciences than are needed to address the major scientific questions of today. We see developing regional networks, and ultimately a national herbarium network, as one mechanism for increasing interest among such students while building an invaluable research resource. As such, it is too important to delay. We recognize that, as technology develops, new standards will be developed and new technologies become available; that is the nature of technology. The USVH organization can provide an effective conduit for rapidly sharing the benefits of such developments among all herbaria.

The BISON project should provide the access to herbarium records and tools for working with them that were part of the original vision for the US Virtual Herbarium project, at least so far as the US is concerned. It is, however, dependent on the quantity and quality of records made available to it. The USVH project’s primary focus is on helping herbaria both provide the needed records and ensure that are of the quality standards needed for use in environmental analyses. In doing so, the project will expand the number of individuals who understand the concepts involved and enable interested individuals to obtain data as it becomes available. Moreover, making information available now has resulted in the herbaria involved receiving feedback concerning some of their specimens, feedback that comes from knowledgeable individuals and will, ultimately, benefit BISON.

## Future directions

Much has been learned about building a herbarium data layer in the US but the majority of herbaria are still not contributing to its development. There are some herbaria that, although digitizing their specimens, do not make the resulting resources available other than on their own network and some that have not started any part of the digitization process. In the latter cases, the problem may be that the herbarium forms a very small part of the responsibilities of the person in charge, or that the person in charge does not know how to start, or that he or she simply does not have the time. Personal contact is often a key step to bringing isolated herbaria into a network. When making such contacts, the benefits that will accrue from membership in a network need to be presented in terms that are relevant to the mission of the herbarium concerned and the person or persons running it. These benefits should, to the maximum extent possible, be immediate and direct. The greatest benefit, without question, is funding but software developments combined with the ability to share resources with and tap into the knowledge of those already in a network have substantially reduced the amount of funding required.

The benefits to medium-sized and smaller herbaria of participating in a regional herbarium include greater publicity, the ability to show how their specimens contribute to overall knowledge, and a mechanism for identifying where to focus future collecting efforts, all of which help validate their worth to institutional administrators. It provides students at academic herbaria an opportunity to participate in a regional and national informatics enterprise while improving the currency of their education. In addition, it helps build professional relationships among individuals who, because of disparate interests and obligations, might not normally connect with each other. Other benefits depend on the resources made available at the network level. These need to benefit a wide range of individuals because it is by offering such benefits that herbaria, and collections in general, earn public support. Such tools can range from quizzes about plants in a grocery store, to games where participants score points for being able to identify plants from images.

Investment in medium-sized and smaller herbaria can have major impacts on the botanical sciences in the US. These herbaria, their associated curatorial staff and users often provide the experiences that steer students towards the botanical sciences. This is important because a disproportionate number of graduate students come from such institutions. Research intensive universities, state and federal agencies, and non-government organizations are dependent upon these “feeder institutions” to provide a flow of graduate students and professional botanists.

All larger herbaria are digitizing their collections, usually maintaining their own database and web site in addition to participating in one or more networks. If, as is the case in several large herbaria, much of their current research and collection activity lies outside the US, these activities may be most appreciated outside the US but they are essential to attainment of the US Virtual Herbarium’s overall goal, digitization of all specimens in all US herbaria. Large herbaria can benefit from joining a network by becoming the “go-to” herbarium for web-related resources. They are also usually better positioned to attract funding for positions to support a regional network. In addition, contributing records to the region where they are located helps them demonstrate that they are “good neighbors” which may assist them in obtaining benefits from the jurisdiction in which they lie.

An area that still needs improvement is building the bridges needed for sharing ideas, information, and concepts between those directly responsible for herbaria and those with specialized knowledge in areas relating to digitization and use of the flood of information it is providing. There are many such areas: biodiversity informatics, information technology, computer science, geography, and education. Working with specialists in these areas will develop a richness and synergy that benefits all involved. The US Virtual Herbarium project can help extend the benefits of such interactions throughout the herbarium community. Among these benefits are increased efficiency in herbarium management which will, ultimately, free up the time of those involved for research and educational activities. Developing these interactions requires that all involved respect each other’s different backgrounds, obligations, interests, and knowledge.

What of the immediate future? There are several steps that the USVH project plans to take. Regional consortia or networks are extremely beneficial in helping move multiple herbaria forward, but some parts of the country have, as yet, no effective network. One of our immediate targets is to facilitate linking all herbaria to a regional network. This can be accomplished either by expanding the region covered by an existing network, possibly with separate portals for subregions (e.g., SEINet and IRHN), or by creating new networks. Both scenarios will require acquisition of additional server space and support personnel.

Georeferencing vastly increases the value of collection records and enables searches across space which may be more relevant to some research questions than searches across taxa (Johnson et al. 2011). It is an aspect that greatly benefits from collaboration but also helps build the social infrastructure needed for effective collaboration (Constable et al. 2010). US herbaria have not, as yet, implemented collaborative georeferencing although some herbaria have georeferenced a substantial portion of their specimens. In many instances, however, this may mean only that there is a latitude and longitude associated with the record. Such limited data make it possible to obtain a picture of the overall distribution of a taxon but do not satisfy the needs of those engaged in environmental analyses (Chapman and Wirczorek 2006).

Data cleaning is another aspect that has, as yet, received surprisingly little attention from herbarium networks. The primary reason may be that the focus is on obtaining records and engaging herbaria, but there are now enough records in each network that building mechanisms for routinely identifying problems is highly desirable. These should be run at the herbarium level with cleaning at the regional level being a second line of defense. The need is for tools that check that georeference and elevation data are at least consistent with the lowest political unit used (usually county for the US, often state for other countries). The scientific name used must also be checked for accuracy because some herbaria may have recorded data in databases (or spreadsheets) without verifying that the names entered were valid. Another check, one that is probably best combined with georeferencing, is for the spelling of place names. Some will be found to be phonetic renditions (Chian for Cheyenne); others are merely misspellings.

Crowd-sourcing of data capture is already being explored in the US and elsewhere. What is not clear yet is how many volunteers can be found to take a short, online training session and then enter data for herbarium specimens online nor whether it is best to focus on identifying and capturing critical data, leaving capture of the remaining data to a later stage, or whether to try and capture all data at once. As with so many other decisions, there are pros and cons to both approaches. It is important, however, that we are transparent in reporting our accomplishments. Capturing a few fields from a million labels is not the same as capturing all label information from a million records.

Taxpayer funds, whether federal, state, or local, will not cover the cost of digitizing herbaria and maintaining herbarium networks. We must aggressively pursue other funding opportunities, including some that most of us involved with herbaria do not normally approach, such as wealthy individuals with an interest in the environment and stores that sell equipment and clothing to people who enjoy hiking. “We” in this case involves all in charge of herbaria but the approach each person takes has to reflect their abilities and interests and as well of those of the herbarium for which they are responsible. It should also complement their other responsibilities (and conform to their institution’s guidelines). The US Virtual Herbarium project can help by disseminating information about successful approaches, developing templates, and seeking funds that will benefit multiple herbaria or networks.

Requests for financial support are more likely to be well received if it can be demonstrated that they will result in a product that benefits many user groups. To encourage use of the information available through existing herbarium networks, we need to work with K-12 educators to develop units that make use of network associated information while meeting state and national science standards. We must also work with state native plant societies, recognizing their value and asking their assistance in promoting use of our networks and their further development. We also need to make sure that government employees are aware of the information being made available, emphasizing its value in their work and to their constituents. And in all these interactions, we must not forget to ask what would make the resources we are developing more useful.

In addition to seeking funding from new sources, all those involved in herbaria must keep looking at work flows to see if they can be made more efficient. Sometimes simple changes, such as using preprinted barcodes to put a catalog number on a specimen rather than using a stamping machine, can save considerable time, time that can used for other purposes. Another possible change is to enable and expect those who borrow specimens to enter their information into the owner’s database or into a regional database from which the owning herbarium could import the records and images. Since almost anyone borrowing specimens nowadays enters information from them into a database, this would require little additional work for the borrower but would greatly aid the loaning institution.

Sustaining the networks also requires maintaining the integrity of the data over time. The costs of doing so are non-trivial because, as Rosenthal (2011) pointed out, “digital data do not tolerate benign neglect”. The specimens themselves are much more resilient in this regard. Moreover, each herbarium, even those that enter data directly into a network database, should maintain a copy of their data. This has the added advantage of ensuring that there are two copies in different locations. Another approach would be for neighboring regions to mirror each other’s resources. This would increase the server space required by individual regions but in a manner that would be mutually beneficial. Eventually this task will, presumably, fall to iDigBio and BISON but, for now, herbaria and herbarium networks must adopt alternative approaches.

## Conclusions

The number and distribution of herbaria in the US, together with the number of specimens they house, make them a prime resource for research in many different disciplines. Providing access to their information will enable sophisticated analyses at levels of scale, scope and accuracy that are unparalleled in the life sciences. It can also be used to introduce and encourage a fascination with plants, fungi, and algae by students at all levels in ways that incorporate inquiry. Digitizing herbaria will also enable those who work in herbaria more opportunities to study the organisms they love, and their interactions, by increasing the ease with which diverse user groups can access herbarium-based information without assistance from herbarium personnel.

The impediments to achieving the goal of the US Virtual Herbarium project, digitizing all specimens in all US herbaria, are resource-based, but they can be offset by focusing on the human factor. The project is dedicated to unlocking the vast resource represented by herbarium specimens by assisting in development of the human and knowledge infrastructure needed. It is accomplishing this task by linking people, ideas and tools into an integrated whole. Much of this involves extending the tools, knowledge, and resources developed by funded projects to more herbaria by establishing connections among people with the varied skills and interests needed, thereby building an integrated community of people working towards a common goal.

**Note added in proof:** Results of the 2012 herbarium survey are being posted to http://herbarium.usu.edu/SurveyResults.html. It included a question about georeferencing and asked for more details on network connections (see Appendix 3).

## Appendix 1

### Survey of Digitization in US Herbaria – 2011

This shows the questions asked. It is not the original form; that had a lot more blank space. The survey was kept short out of respect for the respondent’s time.

**Measuring Digitization Progress**

Herbarium Code: ________________________________________________

Specimen total (estimate): _________________________________________

Number of specimens databased: ___________________________________

Number of specimens imaged:______________________________________

URL for searching database: _______________________________________

URL of regional node through which data are available: __________________

Other nodes through which your specimen data are available: _______________

**Basic information**

Herbarium Name:________________________________________________

Department:____________________________________________________

Address 1: _____________________________________________________

Address 2: _____________________________________________________

City: ________________________ Zip Code: ________________________

Phone: ________________________________________________________

PO Box:_________________________ Mail Stop:______________________

Lat.:_________________________ Lon.:_____________________________

Name of contact person: ___________________________________________

Email of contact person:___________________________________________

Taxonomic focus:________________________________________________

Geographic focus:________________________________________________

## Appendix 2

### Web Sites

This is a listing of all web sites mentioned in the text and a brief synopsis of their significance to the paper.

Alabama Plant Atlas: Provides information about plants in Alabama, including information derived from several herbarium databases. http://www.floraofalabama.org

Algaebase: AlgaeBase is a database of information on algae that includes terrestrial, marine and freshwater organisms. http://www.algaebase.org

Apiary: Program for enabling capture of collection data in the field. http://www.apiaryproject.org

Atrium: Technology data for managing diverse biodiversity data. http://www.brit.org/explore/bioit

Consortium of California Herbaria: State herbarium network. http://ucjeps.berkeley.edu/consortium

Consortium of North American Bryophyte Herbaria: Taxonomically focused herbarium network. http://symbiota.org/bryophytes/index.php

Consortium of North American Lichen Herbaria. Taxonomically focused herbarium network. http://symbiota.org/nalichens/index.php

Consortium of Pacific Northwest Herbaria: Regionally focused herbarium network. http://www.pnwherbaria.org

Cooperative Taxonomic resource for American Myrtaceae: Taxonomically focused herbarium network.http://cotram.org/collections/index.php

Index fungorum: Synonymized list of fungal names. http://www.indexfungorum.org/names/names.asp

Institute for Museum and Library Services (IMLS): US federal agency that has funded some of the work described. http://www.imls.gov

Intermountain Region Herbarium Network: Regionally focused herbarium network. Shares database with SEINet. http://intermountainbiota.org/portal/index.php

International Plant Names Index (IPNI): List of plant names and an indication of whether or not they are valid. Only shows nomenclatural synonyms. http://www.ipni.org

Mycoportal: Taxonomically focused herbarium network. http://mycoportal.org/portal/index.php

 National Information Management and Support System (NIMSS): Information systems that serves the Agricultural Experiment Stations and the Extension Service in each state. http://nimss.umd.edu

National Science Foundation (NSF): US federal agency that has funded much of the work described. http://www.nsf.gov

SERNEC: Regional network for strengthening communication and promoting data sharing among herbaria, now also serving as a regional herbarium network. http://www.sernec.org

SourceForge: Web site that provides access to open source software. http://sourceforge.net

Southwestern Environmental Information Network (SEINet): Regionally focused herbarium network. Herbaria in the Intermountain Region share data with this network. http://swbiodiversity.org/seinet/index.php

Symbiota: Open source software for promoting collaboration and data sharing among herbaria. http://symbiota.org/tiki/tiki-index.php

Tropicos: Nomenclatural resource for bryophytes and vascular plants that shows how a name has been treated in different publications. Also the specimen database of the Missouri Botanical Garden. http://www.tropicos.org

US Virtual Herbarium (USVH): Project for promoting digitization in US Herbaria. This web site is not being maintained because of funding decisions by the US government. Arrangements are being made to move it, or something similar, to another site. http://usvirtualherbarium.org

Utah State University Herbarium: Provides access to the results of the 2012 herbarium survey. http://herbarium.usu.edu

WisFlora: Provides information about plants in Wisconsin, including information derived from several herbarium databases. http://www.botany.wisc.edu/wisflora.

## Appendix 3

### US Herbarium Survey 2012

Presented below are the questions asked on the 2012 survey. To save space, only the questions asked about digitization are shown. For more information, see http://herbarium.usu.edu/SurveyResults.html

About how many specimens are there in your herbarium? Please provide a single number, not separate estimates for different kinds of specimens.

**Databasing:** Some herbaria are entering data for a few fields when imaging, then completing data entry later. For that reason, there are two questions concerning databasing.

How many specimens in your collection have been at least partially databased?

How many specimens have been fully databased (you may answer unknown)?

**Imaging.** The questions below distinguish between imaging specimens (biological material) and imaging labels. If you do not distinguish between the two, put an asterisk by the answer for specimens.

How many of your *specimens* have been imaged?

How many of your *labels* have been imaged?

**Georeferencing**. How many of your specimens have latitude and longitude information?

#### Access

The next questions ask about the web site(s) through which your specimen information is available. If your database cannot be searched via a web site, you have finished the survey. Thank you for taking the time to complete it. If you wish to make a comment or suggestion, please use the space the end. Hand written comments are welcome

If your records are searchable via an institutional web site, what is its URL?

If your records are searchable via one or more regional websites, what are their URLs?

If your records are searchable via one or more taxonomically focused web sites, what are their URLs?

If you provide searchable access to your records through a regional web site that lies primarily outside the US, please indicate the focus of the site(s) and its(their) URL(s).

YOUR Comments:

## References

[B1] Flora of North America Editorial Committee (1993-present) Flora of North America north of Mexico. Oxford University Press, New York.

[B2] Chapman AD Wieczorek J (Eds) (2006) Biogeomancer, Guide to best practices for georeferencing. Global Biodiversity Information Facility http://www.gbif.org/orc/?doc_id=1288

[B3] Constable H Guralnick R Wieczorek J Spencer C Townsend Peterson A The Vertnet Steering Committee (2010) Vertnet: A new model for biodiversity data sharing. PLoS Biology 8: e1000309. doi: 10.1371/journal.pbio.1000309 PMC2821892 20169109

[B4] Johnson JP (2011) Marauding Moths. The Scientist. http://the-scientist.com/2011/10/01/marauding-moths/

[B5] Johnson KG Brooks SJ Fenberg PB Glover AG James KE Lister AM Michel E Spencer M Todd JA Valsami-Jones E Young JR Stewart JR (2011) Climate change and biosphere response: Unlocking the collections vault. Bioscience 61: 147-153. doi: 10.1525/bio.2011.61.2.10

[B6] Kouwenberg LR McElwain JC Kürschner WW Wagner F Beerling DJ Mayle FE and Visscher H (2003) Stomatal frequency adjustment of four conifer species to historical changes in atmospheric CO_2_. American Journal of Botany 90: 610-619. doi: 10.3732/ajb.90.4.610 21659156

[B7] Kramer AT Zorn-Arnold B Havens, K (2010) Assessing botanical capacity to address grand challenges in the United States. 64 pp. [plus appendices] http://www.bgci.org/usa/bcap

[B8] Legler B (2011) Specimen Imaging Documentation, version 4.0. Consortium of Pacific Northwest Herbaria http://www.pnwherbaria.org/documentation/specimenimaging.php

[B9] Rosenthal DSH (2011) Paying for long-term storage. Presentation at the Coalition for Networked Information Membership Meeting, December 12–13. http://www.youtube.com/watch?&gl=US&hl=en&client=mv-google&feature=m-feedu&v=_5lQxmyz3xY&nomobile=1

[B10] Thiers B (2012+) Index herbariorum: a global directory of public herbaria and associated staff. http://sciweb.nybg.org/science2/IndexHerbariorum.asp

[B11] Woodward FI Bazzaz FA (1988) The response of stomatal density to CO_2_ partial pressure. Journal of Experimental Botany 39: 1771-1781. doi: 10.1093/jxb/39.12.1771

[B12] Zangeri AR Berenbaum MR (2005) Increase in toxicity of an invasive weed after reassociation with its coevolved herbivore. Proceedings of the National Academy of Sciences 102(43): 15529–15532. doi: 10.1073/pnas.0507805102 PMC1266144 16230607

